# Sertoli Cells Are Susceptible to ZIKV Infection in Mouse Testis

**DOI:** 10.3389/fcimb.2017.00272

**Published:** 2017-06-21

**Authors:** Zi-Yang Sheng, Na Gao, Zhao-Yang Wang, Xiao-Yun Cui, De-Shan Zhou, Dong-Ying Fan, Hui Chen, Pei-Gang Wang, Jing An

**Affiliations:** ^1^Department of Microbiology, School of Basic Medical Sciences, Capital Medical UniversityBeijing, China; ^2^Department of Histology and Embryology, School of Basic Medical Sciences, Capital Medical UniversityBeijing, China; ^3^Center of Epilepsy, Beijing Institute for Brain DisordersBeijing, China

**Keywords:** Zika virus, Dengue virus, testis, sertoli cells, tight junctions, sperm cells, AG6 mouse

## Abstract

Flaviviruses including Dengue virus (DENV), Yellow fever virus (YFV), West Nile virus (WNV), and Japanese encephalitis virus (JEV) are global health problems that caused several serious diseases such as fever, hemorrhagic fever, and encephalitis in the past century. Recently, Zika virus (ZIKV) which spreads from Asia to American and causes millions of infections emerges as a new dangerous member of the genus of *Flavivirus*. Unlike other well-known flaviviruses, ZIKV can be transmitted sexually and infect testes in murine models. Its impacts on sperm functions, and the exact susceptible cells, however, are not entirely clear. To investigate these issues, we infected interferon α/β and γ receptors deficient AG6 mice with ZIKV and examined the outcomes of infection using an assortment of physiological, histopathological, immunological, and virological techniques. We found that infected mice displayed signs of reproductive system disorder, altered androgen levels in serum, and high viral load in semen and testes. Additionally, histopathological examinations revealed marked atrophy of seminiferous tubules and significant reduction in lumen size. Notably, these were accompanied by positive staining of ZIKV antigens on sertoli cells, detection of viral particles and vacuole changes within cytoplasm of sertoli cells. The susceptibility of sertoli cells to ZIKV was further validated *in vitro* study using cell lines. Importantly, the disruption of tight junctions within testis and altered sperm morphology were also observed in ZIKV infected mice. It is well-known that tight junctions formed by adjacent sertoli cells are major component of blood testis barrier, which plays important roles in maintenance of microenvironment for spermagenesis in testis. Taken together, these results demonstrate that sertoli cells are susceptible to ZIKV infection, which results in the disruption of tight junctions in testis and causes abnormal spermatogenesis in mice. These results also imply that long-term impact of ZIKV infection on human male reproductive system requires close monitoring.

## Introduction

Zika virus (ZIKV) is an arbovirus (arthropod-borne virus), and belongs the *Flavivirus* genus within the *Flaviviridae* family, which are small enveloped single positive stranded RNA viruses including a number of human pathogens such as Dengue virus (DENV), Yellow fever virus (YFV), West Nile virus (WNV), and Japanese encephalitis virus (JEV). ZIKV was first identified in a sentinel monkey in Uganda in 1947 and first isolated in human in 1952 (Vasilakis and Weaver, 2016; Wikan and Smith, 2016). Following a long dormant period, sporadic outbreaks of human ZIKV infections were reported in Pakistan and Malaysia in 1977. A larger ZIKV outbreak occurred in the Pacific islands in 2007 with more than half of the island population infected. More recently in 2015–2016, ZIKV stormed Latin America, infected several million people in Brazil and Columbia, and rapidly spread to USA, Southeast Asia and other 70 countries/territories, made World Health Organization (WHO) to declare it as a Global Emergency in February 2016 (Lessler et al., 2016; Ribeiro et al., 2016).

Historically, ZIKV was neglected because ~80% of infected adult patients are asymptomatic and the 20% of symptomatic cases generally experience a non-specific, mild, and self-limiting febrile illness (Petersen et al., 2016). However, ZIKV has raised major concern recently due to its rapid dissemination and close association with microcephaly in children, Gullian-Barr syndrome in adults (Lazear et al., 2016; Lessler et al., 2016; Petersen et al., 2016; Wikan and Smith, 2016). Most unusual for a flavivirus, in addition to transmission by mosquitoes, ZIKV can be transmitted sexually (D'Ortenzio et al., 2016; Tang et al., 2016). There have been 17 reported male-to-female (D'Ortenzio et al., 2016) and male-to-male (Deckard et al., 2016) transmission globally (Brooks et al., 2016; World Health Organization, 2016). ZIKV could be detected in semen as late as 188 days after the onset of infection (Nicastri et al., 2016). This raised urgent need to elucidate several questions: (1) what type of cells in testes supports viral persistence? (2) what are the structural and functional impacts of ZIKV infection on testes? And, what molecular and cell biological mechanisms were involved during ZIKV infection of testes?

Testes are the major male reproductive organs in which sperms are produced (spermatogenesis), and androgens, primarily testosterone, are synthesized. Testis consists of two separate compartments: seminiferous tubules and interstitial spaces. In seminiferous tubules, there were spermatic cells at different stages of spermatogenesis and sertoli cells, which embrace spermatogenic cells and form the seminiferous epithelium with them, and peritubular myoid cells that form the surrounding of the seminiferous tubules (Mruk and Cheng, 2015; Han et al., 2016). The interstitial compartment is mainly comprised of androgen producing Leydig cells, a majority of the interstitial cell populations, and some macrophages, which constitutes ~20% of the interstitial cells in rodent (Chen et al., 2009). Testis is a typical immunoprivileged organ which protects sperm cells from immune attack (Han et al., 2016). Immune cells and factors normally cannot pass from the blood to the lumen of a seminiferous tubule due to the presence of blood testis barrier (BTB), whose major components are tight junctions (TJs), which is believed to form by adjacent sertoli cells (Johnson et al., 2008). Occludin (Ocln), various members of claudin (Cldn) and ZO-1, as main TJs-associated proteins, are thought to be directly involved in this barrier mechanism (Tsukita and Furuse, 1998; Chung et al., 2001), and play an important role in the maintenance of testicular homeostasis. Therefore, sertoli cells have always been depicted as critical cells for spermatogenesis (Chen and Liu, 2015). How ZIKV infection affects sertoli cells thus becomes a significant question in pathogenesis.

To address this question, in the current work, we challenged interferon α/β and γ receptors deficient AG6 mice with ZIKV and monitored the outcome of infection in mouse testes. Our data revealed that sertoli cells were susceptible for ZIKV, and the infection leads to the disruption of TJs, abnormal morphological architecture and dysfunction of testes, and disorder signs on male reproductive system, suggesting testes damage and the possible mechanism underlying disorders of male reproductive system caused by ZIKV.

## Results

### Testes are one of important target organs for ZIKV infection in AG6 mice

**Figure 1:**
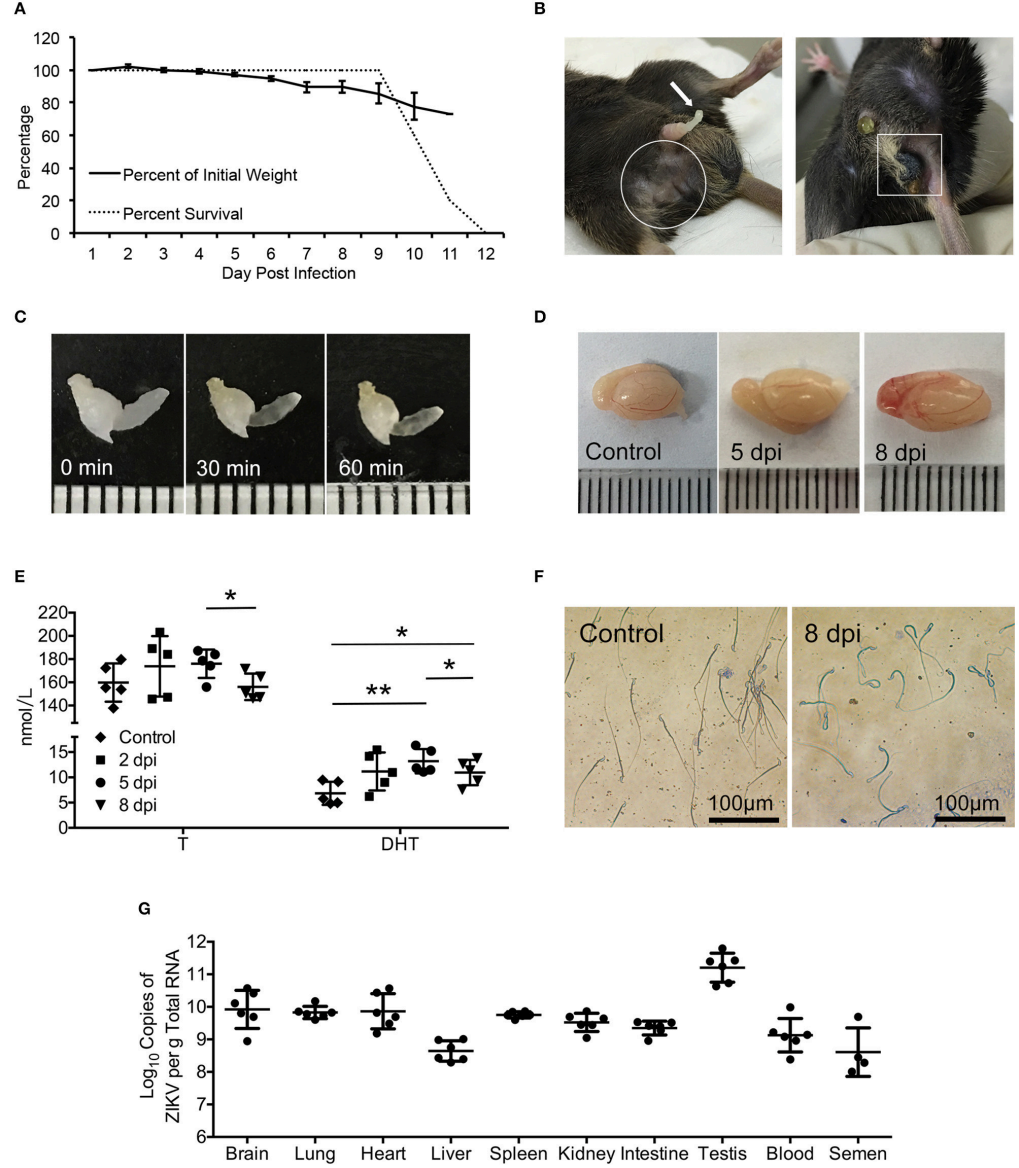
Signs of the disease, changes of serum androgen level and morphology of sperms as well as the virus detection in ZIKV-infected AG6 mice. **(A)** Changes of body weights and survival rate post infected with 10^5^ pfu ZIKV in male AG6 mice (*n* = 5). **(B)** Clinical signs of reproductive system disorder were observed in male ZIKV-infected mice. Spermatorrhea (left, arrow), trichomadesis (left, in frame), and necrosis of scrotum (right, in frame). **(C)** Semen harvested from mice with spermatorrhea at 8 dpi was incubated at 37°C for 30 and 60 min, showing non-liquefied semen. **(D)** Representative image of testis dissected from control and ZIKV-infected mouse at indicated time points. **(E)** Changes of testosterone (T) and dihydrotestosterone (DHT) levels in serum at indicated time points after ZIKV infection. Data are represented as mean ± *SD* (^*^, *p* < 0.05; ^**^, *p* < 0.01) (*n* = 5). **(F)** Morphology of sperms from ZIKV-infected mice at 8 dpi or control mice revealed by Papanicolaou staining. **(G)** Viral load determined by qRT-PCR in organs, serum and semen at 5 dpi. The standard curve was set up based on ZIKV RNA transcribed *in vitro* (*n* = 4–6).

To characterize ZIKV infection in mouse testes, interferon α/β and γ receptors deficient AG6 adult male mouse (Liu et al., 2016) were inoculated subcutaneously (sc) with 10^5^ pfu ZIKV (strain CAS-ZK01). The infected mice displayed illness symptoms such as ruffled hair, hunched back, bradykinesia from 3 days post-infection (dpi) and gradually lost body weight (Figure 1A). From 5 dpi, signs of reproductive system disorder were observed, including perineum swelling (11/13), spermatorrhea (10/13), alopecia (4/13), and necrosis of scrotum (3/13) (Figure 1B). Non-liquefied semen was observed even after incubation at 37°C for 60 min (Figure 1C), which indicated that there was inflammation and infection in genital tract and closely associate with male infertility although a number of factors contribute to this issue (Du Plessis et al., 2013). All mice died around 11 dpi. Dissected testes from infected mice showed obvious bleeding and swelling in testes at 8 dpi (Figure 1D). Androgen levels in serum showed increased trend at 5 dpi and then decreased at 8 dpi (*p* < 0.05); its main active form, dihydrotestosterone, showed similar change pattern and increased more significantly (*p* < 0.01 for 5 dpi and *p* < 0.05 for 8 dpi, Figure 1E). Accordingly, Papanicolaou staining showed a morphological change in sperm cells overtime after infection: there was no evident change at 5 dpi and sperm tails with obvious curl and shortening were observed at 8 dpi (Figure 1F). High virus titers as measured by qRT-PCR assay were detected in several organs and blood, ranging from 10^9^ to 10^11^ copies/g total RNA; the highest titer was found in testis, followed by brain and the lowest in liver (Figure 1G). Notably, virus RNA was also detected in semen with a titer ~10^8^ copies/g total RNA, suggesting that testis is one of major targets for ZIKV infection.

**Figure 2:**
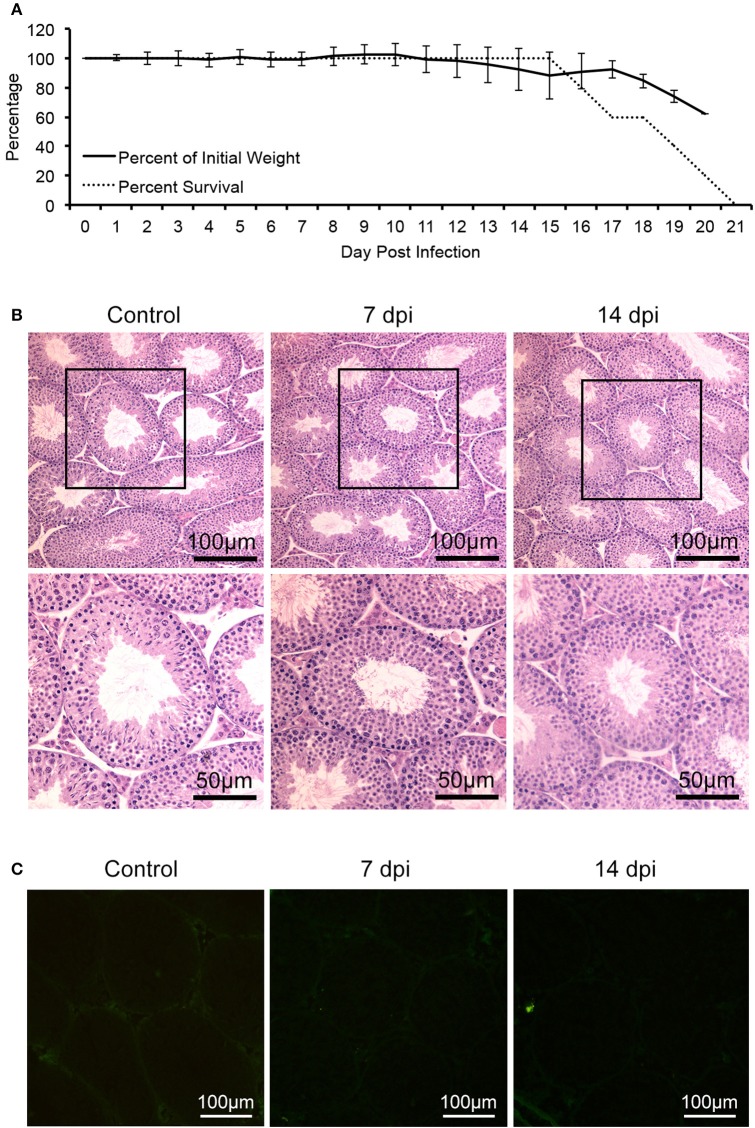
Characterization of AG6 mice challenged with 10^5^ pfu of DENV-2. Control mice were injected with PBS. **(A)** Changes of bodyweights and survival rate after infected with DENV-2 in male AG6 mice (*n* = 5). **(B)** Histopathological changes of testis from DENV-2-infected AG6 mice at different time points as indicated or control mice. **(C)** Analysis of DENV-2 antigens in testis from DENV-2-infected AG6 mice at different time points as indicated or control mice.

In contrast, a control virus, DENV-2 (strain Tr1751), which belongs to flavivirus and has close phylogenetic relationship with ZIKV, despite also capable of infection and causing mortality of the same mice (Figure 2A), produced neither any clinical signs of reproductive system disorder nor evidence of infection of mouse testis (Figures 2B,C), indicating a specific tropism of ZIKV to testes.

### ZIKV infection results in histopathological and transcriptome changes in testis

**Figure 3:**
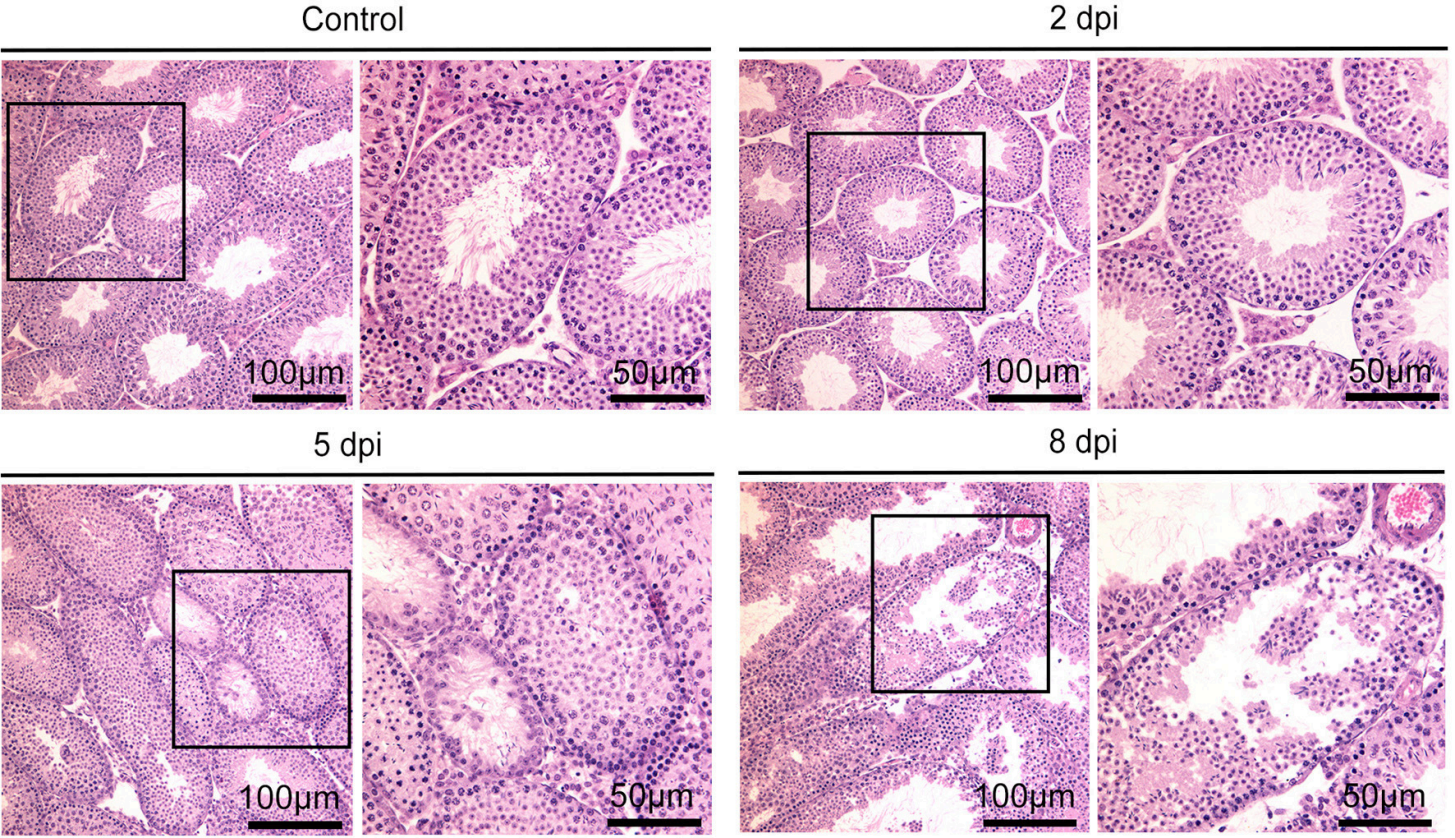
Histopathological changes of testis in ZIKV-infected mice at different time points as indicated or control mice.

Having established that ZIKV can infect mouse testes, we first investigated the histopathological changes in testis of infected mice using Hematoxylin and eosin (H&E) staining. As shown in Figure 3, the interstitial regions between seminiferous tubules began to expand at 2 dpi; with more prominent expansion and observation of proliferated cells at 5 dpi, without obvious infiltration of inflammatory cells. At 8 dpi, there was more obvious seminiferous tubule atrophy and disappearance of lumen. The seminiferous tubules arranged disorderly and the cell layers became thinner. These indicated damage of testes caused by ZIKV infection.

**Figure 4:**
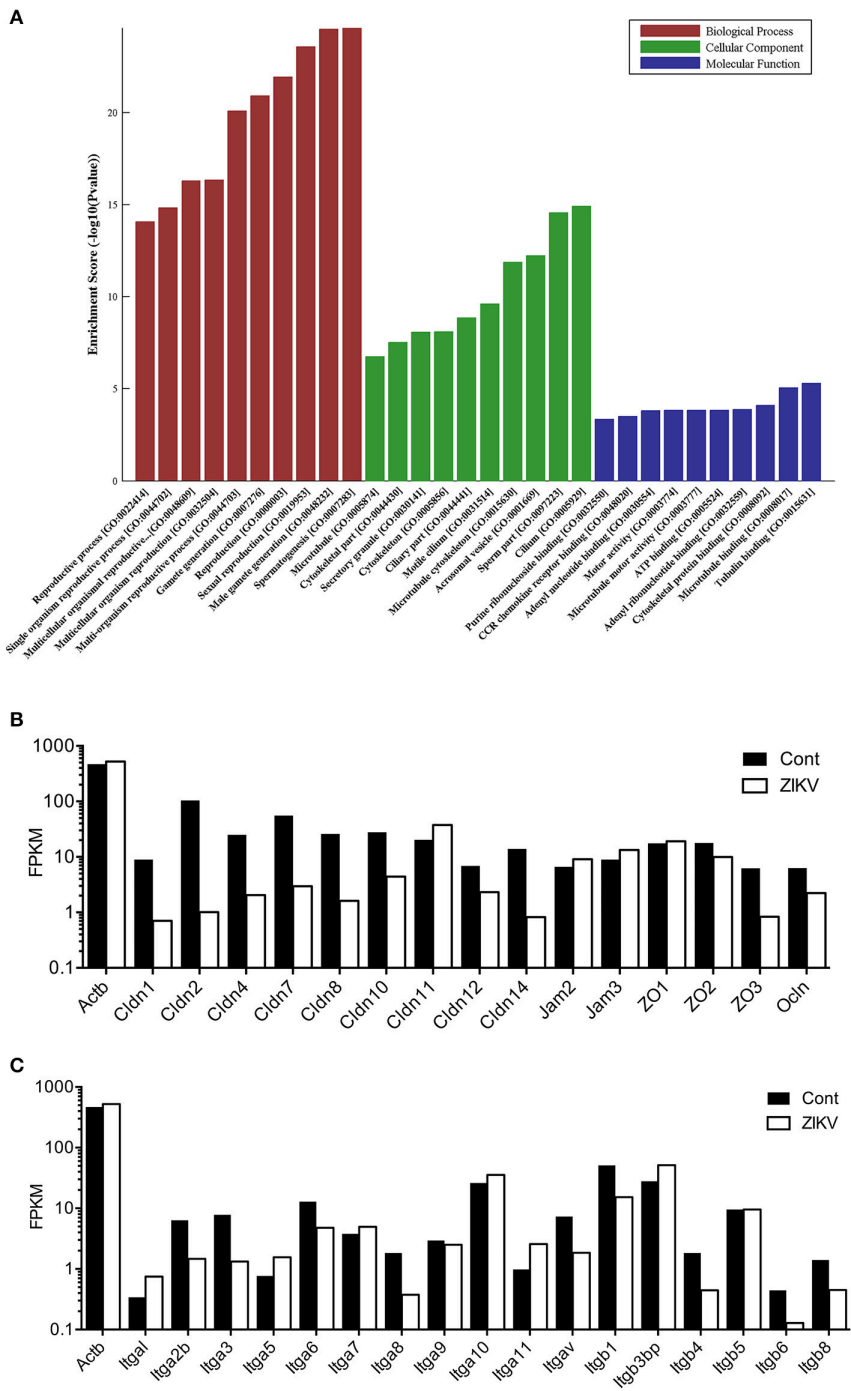
Transcriptome analysis for various pathways in mouse testes at 5 dpi after ZIKV-infection. **(A)** Gene ontology (GO) analysis for gene upregulated in testes of ZIKV-infected mice at 5 dpi (*n* = 3). Uninfected mouse testes serve as control (*n* = 3). The ontology covers three domains: Biological Process (red), Cellular Component (green) and Molecular Function (blue). Fisher's exact test is used for the GO analysis (*p* < 0.05). **(B,C)** Transcriptome analysis of selected genes associated with tight junction pathway **(B)** and integrins **(C)** in ZIKV-infected testes at 5 dpi. The results were expressed as Fragments Per Kilobase Million (FPKM). Beta-actin (Actb) was used as control.

To understanding the global effect caused by ZIKV infection on testes, gene expression profiling was performed. Testes isolated from ZIKV infected mice at 5 dpi or control mice were subjected to transcriptome analysis. In all biological process in testis, spermagenesis showed the most obvious change upon ZIKV infection, as indicated by the gene ontology (GO) analysis (Figure 4A). The cellular component in sperm part almost showed the most obvious change too (Figure 4A). The upregulation of gene associated with spermagenesis, thus indicated an abnormal developing process of germ cells (Figure 4A). The genes associated with TJs and integrin were downregulated in ZIKV infected mice, indicating the altered testicular homeostasis (Figures 4B,C). Taken together, ZIKV infection may have altered the morphological architecture and function of testis.

### ZIKV antigen was mainly distributed in sertoli and macrophage cells in testis

**Figure 5:**
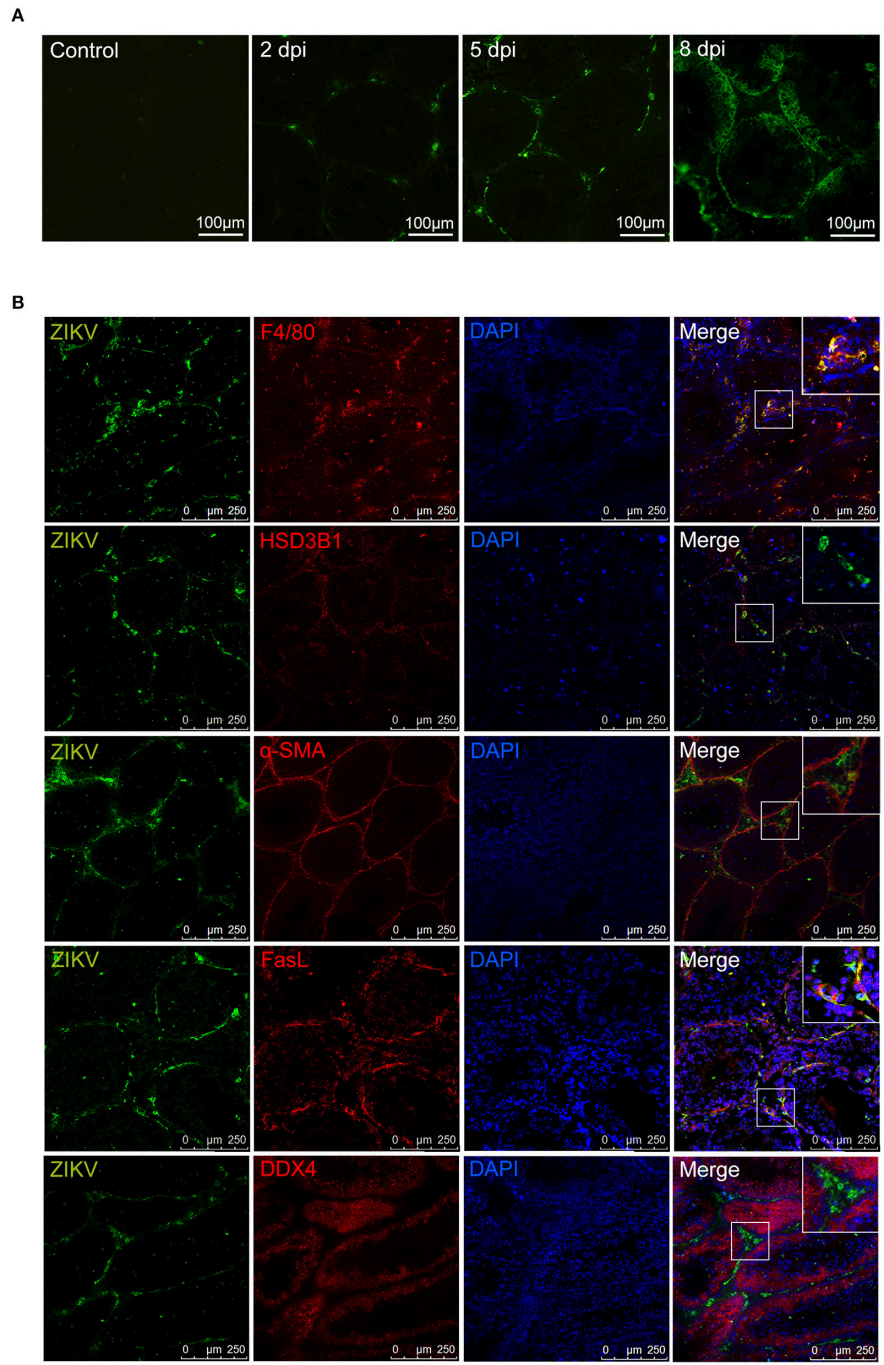
Immunofluorescence staining for detection of ZIKV antigen in mouse testes. **(A)** Distribution of ZIKV antigens in testis at different time points as indicated. **(B)** The co-localization of ZIKV antigens with cells in ZIKV-infected testes at 5 dpi (anti-ZIKV polyclonal antibodies, in green), various cell markers in testis (in red, F4/80 for macrophages, HSD3B1 for Leydig cells, α-SMA for peritubular myoid cells, FasL for sertoli cells, DDX4 for germ cells), and staining for nuclei (DAPI, in blue).

Distribution of ZIKV antigen was analyzed by immuno-fluorescent staining assay (IFA). IFA showed that ZIKV antigens distributed mainly beneath the basement membrane of seminiferous tubules and interstitial spaces. With the progression of infection, the intensity of ZIKV antigens increased and gradually diffused into seminiferous tubules (Figure 5A).

To identify the specific target cells for ZIKV infection in testis, double staining was further performed. In the seminiferous tubules, viral antigen-positive cells mostly co-localized with marker of sertoli cell (FasL) (Bellgrau et al., 1995; Zhang et al., 2013) but not with marker of cells at any stage of spermatogenesis (DDX4). In interstitial spaces, almost all viral antigen positive cells were co-localization with the marker of macrophages (F4/80), not with Leydig cells (HSD3B1) or myoid peritubular cells (anti-α-SMA). These results indicated that sertoli cells and macrophages are susceptible for ZIKV infection in testis (Figure 5B).

**Figure 6:**
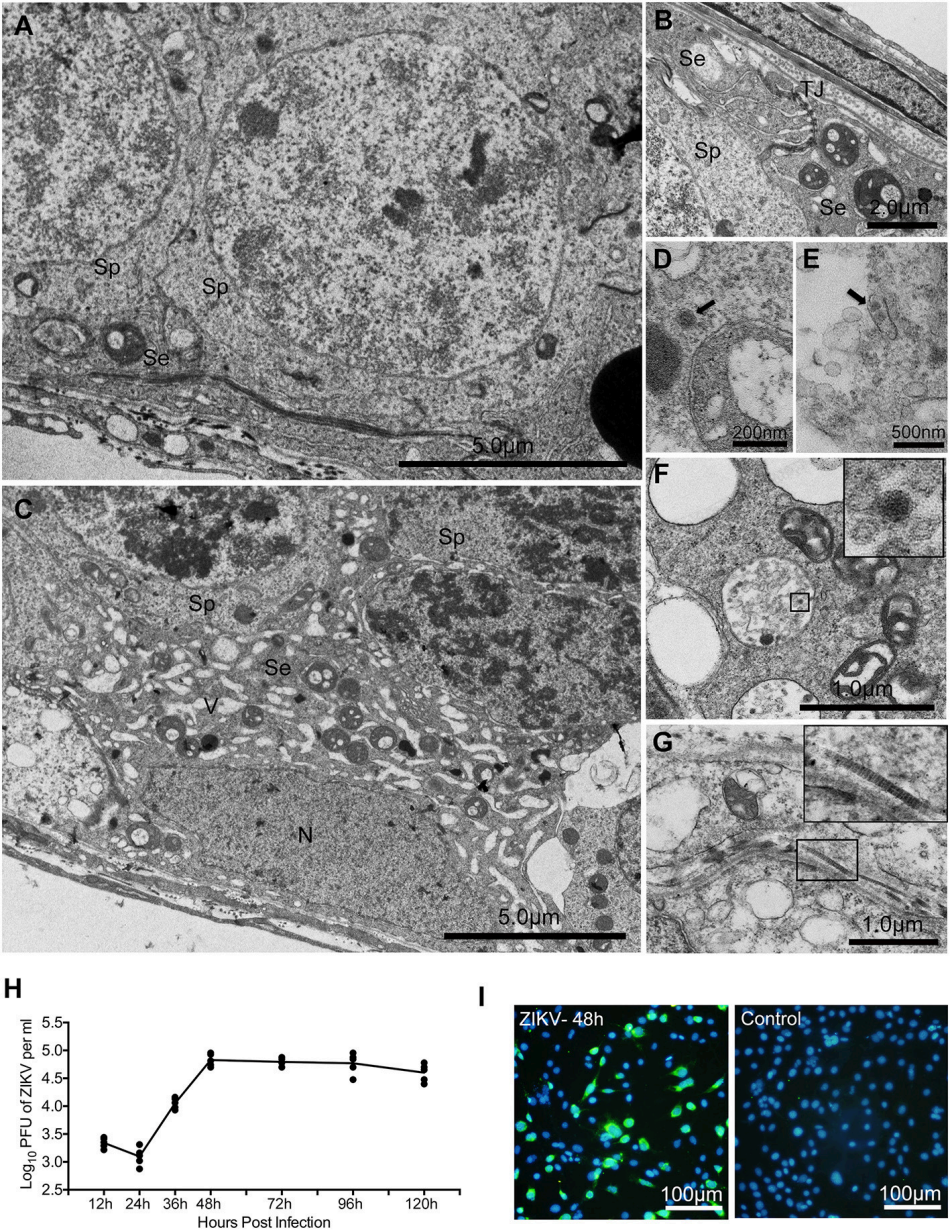
The ultrastructure morphological changes of testes from mice with or without ZIKV infection at 5 dpi under transmission electronic microscopy and replication of ZIKV in sertoli cell line. **(A)** Normal sertoli cell. **(B)** Tight junctions of normal sertoli cells. **(C)** Vacuoles change in ZIKV-infected sertoli cells at 5 dpi. **(D–F)** Viral particles in the cytoplasm of infected sertoli cells at 5 dpi (arrows or in frame). **(G)** Collagen fibers in seminiferous tubules. **(H)** Sertoli cell line was infected by ZIKV (multiplicity of infection, MOI = 1). Culture supernatant was harvested at different time point as indicated for determining viral titers by plaque assay (*n* = 5). **(I)** ZIKV antigen were detected by immunofluoresecent staining with anti-ZIKV polyclonal antibody at 2 dpi. Se, sertoli cells; Sp, spermatocytes; N, nucleus; TJ, tight junctions; V, vacuoles; CF, collagen fiber.

To pinpoint the detailed morphological changes, the impact of ZIKV infection on sertoli cells was further examined using transmission electron microscope (TEM). In control mouse testis, there were numerous mitochondria with typical tubular cristae in the cytoplasma of sertoli cells that surround sperm cells, and TJs typified by “kisses” contact between sertoli cell plasma membranes were also often observed (Figures 6A,B). In contrast, a large number of vacuoles appeared in the cytoplasm of sertoli cells obtained from infected testis (Figure 6C); notably, virus particles were also observed in the cytoplasm of these cells (Figures 6D–F). Additionally, collagen fibers that rarely found in normal testis appeared frequently in ZIKV infected testis (Figure 6G).

To further verify that sertoli cells are indeed susceptible to ZIKV infection, viral replicative kinetics was examined with a sertoli cell line. There was no obvious virus replication during 24 h after inoculation. The viral titer increased at 36 h post infection, reached a peak level of 10^5^ pfu/ml at 48 h, then maintained at this level until 96 h and showed decreased trend at 120 h (Figure 6H). About 40% cells were positively stained by anti-ZIKV polyclonal antibody at 48 h post infection (Figure 6I). All these results are consistent with the above *in vivo* observations.

### ZIKV infection induced disruption of tight junctions in testes

**Figure 7:**
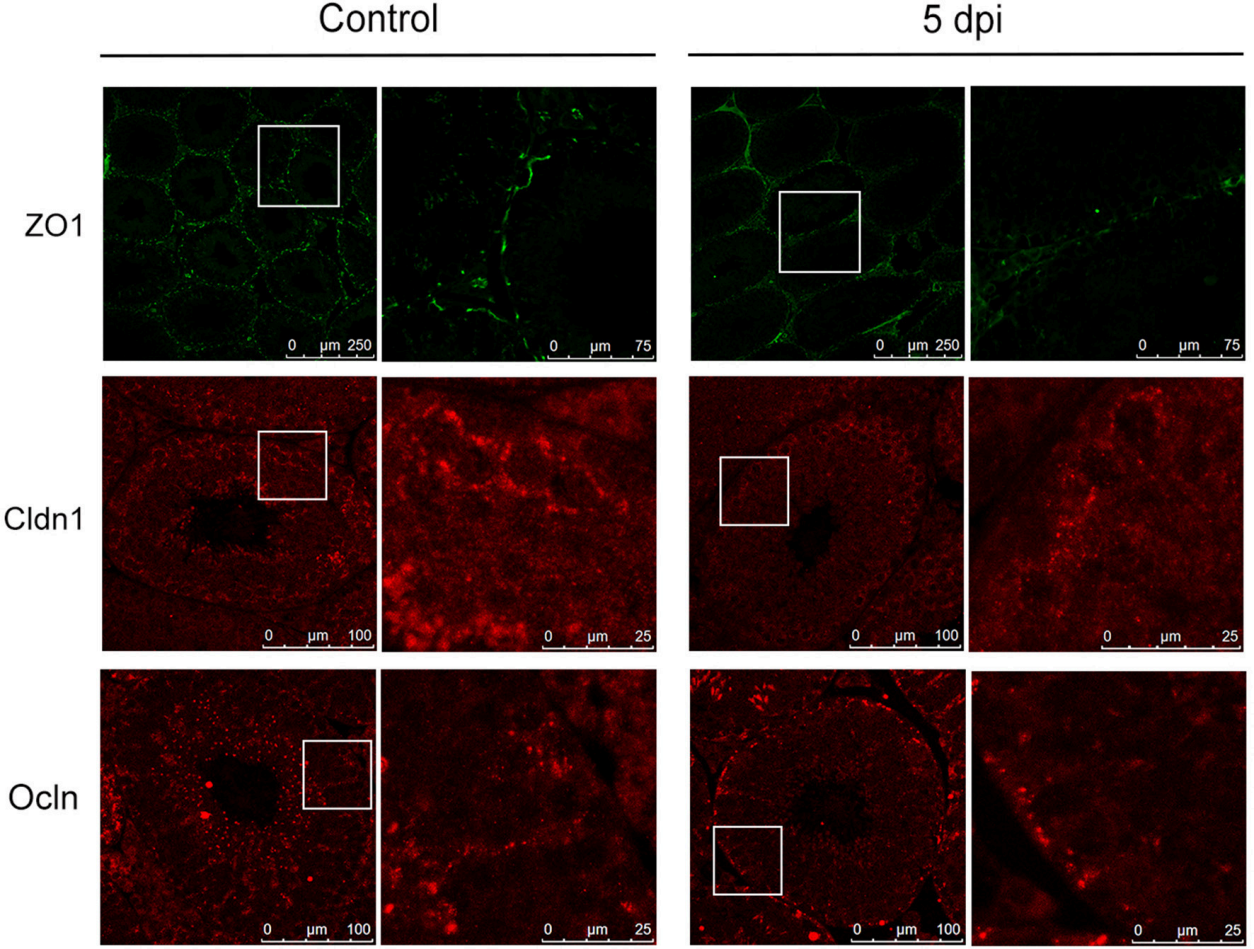
Expression of tight junction associated proteins in testes from mice with or without ZIKV infection. The altered distribution and expression of ZO-1, Cldn1, Ocln in ZIKV-infected testis was analyzed with immunofluorescence staining at 5 dpi compared with control mouse.

In the transcriptome analysis, we found that ZIKV infection markedly downregulated the expression of Ocln and various members of Cldn family, while having had no obvious influence on ZO-1, in comparison to control mice (Figure 4B). Therefore, we examined the expression of these proteins in testes to evaluate whether ZIKV infection caused a disruption of the TJs. In control mouse testis, ZO-1 was highly concentrated and localized at the basolateral site and weakly positive stained in cell-cell border of sertoli cells, while Cldn-1 mainly expressed around germ cells and weakly expressed in apexes of the seminiferous epithelium. Ocln was highly expressed along the cell-cell border of sertoli cells and the basolateral site at liner fashion. After infection, the intensity of immune staining of all above-mentioned TJs-associated proteins gradually reduced overtime. There were no obvious change at 2 dpi and markedly down-expressed, even disappeared in some area at 5 (Figure 7) and 8 dpi. Among three proteins, the most obvious changes occurred in the Ocln distribution after ZIKV infection: the linear TJs along the sertoli cell borders became short and its staining became weaker, and Ocln at the basolateral site were observed in spot fashion (Figure 7). Coincidently, the TJs that often observed in normal testes rarely appeared in infected mouse testes. The histological and molecular biological data indicated that ZIKV infection caused disruption of TJs, which might associate with altered the quality of sperm cells.

## Discussion

Detection of infectious ZIKV in the semen of convalescent men provides a virologic basis for its sexual transmission, which had been observed clinically. In mouse models, it has recently been shown damages of testes after experimental ZIKV infection (Govero et al., 2016; Ma et al., 2016). The exact target cells and the cellular and molecular basis of harms done by ZIKV infection remain to be elucidated. To clarify this issue, we have characterized ZIKV infection in mouse testes using interferon α/β and γ receptors deficient AG6 mouse. Upon ZIKV infection, mice showed obvious illness symptoms and died eventually. Notably, both clinic observation and gross anatomy revealed marked signs of reproductive system disorder such as spermatorrhea and hemorrhage in testis. Additionally, high viral loads were detected in semen and testes among multiple tissues and organs examined. For comparison, a closely related flavivirus, DENV-2, although caused similar systemic symptoms of infection and also mortality, it resulted in neither change in the perineum nor detection of viral antigen in the testis. By H&E staining, atrophic seminiferous tubules and disappearance of lumen were observed, companied by alteration of the serum androgen levels. Moreover, genes associated with TJs and integrins were downregulated, and those related to spermagenesis and extracellular matrix were upregulated in testis. At the protein level, the disruption of TJs and increase of fibro fiber were confirmed by IFA assay and TEM observation. These results indicated that testis is an important target organ for ZIKV infection.

Next, we asked what type of cells in testis is sensitive to ZIKV infection. Testis is the major organ for male reproductive system and consists of multiple cell types. There were spermatic cells at different stages of spermatogenesis, sertoli cells in seminiferous tubules, myoid peritubular cells which surround the tubules and basal lamina. The interstitial compartment is mainly composed of androgen producing Leydig cells and macrophages, which are the second largest interstitial cell populations. In this study, IFA showed ZIKV antigens distributed in both of seminiferous tubules and interstitial spaces, and they increased with the progression of infection. By double staining with antibodies against various cell markers and ZIKV, we found that nearly all viral antigen positive cells in interstitial spaces were co-localization with the marker of macrophages (F4/80), not with Leydig cells (HSD3B1), or myoid peritubular cells (α-SMA). In seminiferous tubules, ZIKV antigen-positive cells often co-localized with the marker of sertoli cells (FasL) but not with the marker of germ cells (DDX4). The susceptibility of sertoli cells to ZIKV were further confirmed by TEM observation, which showed viral particles in cytoplasm of sertoli cells companied by vacuolus change, and *in vitro* study using sertoli cell line, which effectively supported ZIKV replication. Macrophages have been reported to be sensitive to DENV and other viruses (Paria et al., 1996; Wu et al., 2000; Kyle et al., 2007; Balsitis et al., 2009; Prestwood et al., 2012), implying they may be commonly susceptible for various flaviviruses. Taken together, the results indicated that sertoli cells might be major targets for ZIKV in mouse testes.

Because main function of sertoli cells is to nourish the developing sperm cells through the stages of spermatogenesis, they were described as “mother cells” or “nurse cells” when they were discovered (Chen and Liu, 2015). Sertoli cells are the only somatic cells that constitute the seminiferous epithelium with developing germ cells. The spermatogonia locate in the basal compartment (deep to the level of the TJs) and the more mature forms embraced by columnar sertoli cells migrate to the adluminal compartment. The TJs between adjacent sertoli cells are comprised of a number of proteins and play an important role in the maintenance of essential homeostasis for spermatogenesis. Of these, the major types are the Cldn and Ocln, which associate with different peripheral membrane proteins such as ZO-1 located inside the plasma membrane. As the TJs adjacent sertoli cells are essential in maintaining microenvironment of spermatogenesis, we speculated that ZIKV replication in sertoli cells may disrupt TJs and then influence the quality of sperm cell. As expected, in this study, there were vacuolar changes in sertoli cells observed under TEM and downregulated expression of TJs-associated proteins such as various members of Cldn family and Ocln at RNA and protein levels in testis after ZIKV infection, indicating that there might be a decreased synthesis of TJs-associated proteins caused by damaged sertoli cells, which closely linked to reduced integrity of TJs seen after ZIKV infection. Accordingly, altered morphology of sperm cells and increased expression of spermatogenesis-associated genes were also observed. In combination with reported clinical signs of male reproductive disorder in patients with ZIKV infection (Foy et al., 2011; Musso et al., 2015; Heuvelings et al., 2017), the results indicated that ZIKV infected sertoli cells and damaged the TJs, which could compromise the function of BTB and alter spermatogenesis process and then affect quality of sperm cells. The functional and structural changes in testis not only helped the sexual transmission of ZIKV, but also had potential impact on spermatogenesis. Therefore, other than elucidating a potential mechanism of sexual transmission ZIKV, our study also highlights monitoring long-term impact caused by this virus on male reproductive system.

There are very limited reports regarding ZIKV infection in testes. During the preparation of our manuscript, two groups separately published their results on ZIKV infection in reproductive system of male mice. Govero et al. challenged mice with ZIKV after treatment with interferon α/β receptor (IFNAR) neutralization antibody, and found that the testis was damaged by viral replication. Ma et al. used the IFNAR deficient A129 mice as an infection model and also found that the testis was damaged by ZIKV infection. In agreement with these findings in either wild type mice with IFNAR neutralization antibody IFNAR deficient mice, we demonstrated that in interferon α/β and γ receptors knock-out mice, ZIKV infection also caused damage to testes.

Other than the aforementioned similarity, however, we also made other observations that are not entirely the same with those two published papers. One is regarding specific target cells. Govero et al. indicated that sertoli cells may be the key target but without direct evidence while Ma et al. observed ZIKV antigen in peritubular myoid cells and spermatogonium cells. We found, through several experimental approaches, ZIKV virus only infected sertoli cells and macrophages. The lack of detection of ZIKV antigen in other testicular cell types might associate with the use of different mouse models (wild type vs. A129 vs. AG6), and different sampling times post-ZIKV infection. It is theoretically plausible that ZIKV may infect some types of testicular cells at an early time-point, but infect other types at a later time-point. More investigations are required to resolve these differences in details.

In summary, this study demonstrated that testis is an important target organ for ZIKV in interferon α/β and γ receptors deficient AG6 mice, and that sertoli cells are susceptible to ZIKV. Furthermore, ZIKV infection of sertoli cells damaged TJs and spermatogenesis in mice. These results provide biological basis to explain clinical observations that some ZIKV-infected humans showed genitourinary symptoms such as hematospermia, dysuria and perineal pain (Foy et al., 2011; Musso et al., 2015; Heuvelings et al., 2017). Our study also highlights the importance of long-term monitoring on male reproductive system in ZIKV infected men.

## Experimental procedures

### Cells and viruses

Vero cells (African green monkey kidney cells) were regularly maintained in minimum essential medium (MEM, Gibco, USA) supplemented with 5% fetal bovine serum (FBS, PAN, Germany) at 37°C. C6/36 cells (Aedes albopictus cells) were regularly maintained at 28°C in RPMI 1640 (Gibco, USA) supplemented with 10% FBS. Sertoli cell line was purchased from China Infrastruture of Cell Line Resources and regularly maintained in Dulbecco's Modified Eagle Medium (DMEM, Gibco, USA) supplemented with 10% FBS at 32°C.

Asian ZIKV (strain CAS-ZK01) was kindly provided by Dr. George F. Gao (Institute of Microbiology, Chinese Academy of Sciences, Beijing, China). Dengue virus serotype 2 (DENV-2, strain Tr1751) was laboratory-stored. The virus was propagated in C6/36 cells and determined the titers by plaque assay on Vero cells with 1.2% methylcellulose. Stocks were stored at −80°C until use.

### Ethics statement

All the animal experimental procedures were conducted in reviewed and approved by the Experimental Animal Welfare and Ethics Committee of Tsinghua University, Beijing, China and the animal ethics committees of Capital Medical University, Beijing, China

### Mouse experiments

C57BL/6 mice deficient in interferon α/β and γ receptors (AG6 mouse) were purchased from Institut Pasteur of Shanghai, Chinese Academy of Sciences. The mice were bred and maintained under a specific pathogen-free animal facility at Tsinghua University.

For ZIKV infection, 8–10 week-old male AG6 mice were challenged with 10^5^ pfu ZIKV through subcutaneous (sc) injection. Mice infected with 10^5^ pfu of DENV-2 through sc or administered with phosphate buffered saline (PBS) serve as control. Survival indexes, including body weight, disease symptoms and survival rates of mice were recorded daily till death. Sera and organs of infected mice were sampled at 2, 5, 8 day post-infection (dpi) for determination of androgen, virus detection, and histological examination.

Meanwhile, testes from mice with ZIKV infection were harvested at 5 dpi and subjected to KangChen Bio-tech. Inc. (Shanghai, China) for transcriptome analysis. Uninfected mice serve as control. There were three mice in each group.

### ZIKV mRNA quantification

The infected and control mice were euthanized by cervical dislocation at different time points as indicated and major organs were harvested and homogenated in Trizol (Transgen, China, ET101-01). RNA was isolated from tissue lysates according to manufacturer protocol. A pair of primers (forward: 5′-TCAGACTGCGACAGTTCGAGT-3′; reverse: 5′-GCATATTGACAATCCGGAAT-3′) was designed to detect ZIKV mRNA. Real-time qPCR analyses were performed with Quant One Step qRT-PCR (Tiangen, China, P4127) on 7,500 Real Time PCR System (Applied Biosystems, USA). Quantification of the copies of ZIKV mRNA was determined by standard curve method. ZIKV genome RNA transcripted *in vitro*, kindly provided by Prof. Ai-hua Zheng from CAS, was quantified and used as standard template to establish the standard curve.

### Determination of androgen in serum

Mouse serum was sampled at 2, 5, 8 dpi and stored at −80°C after harvested. Concentrations of Testosterone (T) and Dihydrotestosterone (DHT) in serum were determined by ELISA kits separately (T: AMEKO, China, AE90626Mu; DHT: AMEKO, China, AE90941Mu) according to as manufacturer protocol based on regular indirect ELISA detection. In brief, 50 μl of serum were added to each well-coated by antibodies to androgen and incubated at 4°C overnight. After washing with PBS, biotinylated-seconary antibodies and streptavidin- horseradish peroxidase (HRP) were added and incubated at 37°C for 60 min. After washing and color development, absorbance was read at 450 nm in multiskan spectrum 1500 (Thermo, USA).

### Immunofluorescence staining

For analysis of viral antigens in testis *in situ*, ZIKV-infected mice were euthanized by cervical dislocation at different time point as indicated. Testes were quick-frozen immediately in optimal cutting temperature compound (OCT, SAKURA, USA) in liquid nitrogen to prepare frozen sections with freezing-microtome (Leica, Germany). For analysis of ZIKV antigens and their locations, immunofluorescence double staining was conducted. Rabbit anti-mouse FasL (1:300; Abcam, AB15285), rabbit anti-mouse HSD3B1 (1:300; Abcam, AB65156), rabbit anti-mouse α-SMA (1:300; Santa Cruz, AB1563153), Rabbit anti-mouse DDX4 (1:500, Abcam, AB13840), and rabbit anti-mouse F4/80 (1:100; Abcam, AB111101) as well as mouse anti-ZIKV polyclonal serum (1:50) were applied as primary antibodies and incubated with the testis sections respectively at 4°C overnight. Alexa Fluor® 594 donkey anti-rabbit IgG (H+L) (1:400; A21207, Life technologies, USA) and Alexa Fluor® 488 goat anti-mouse IgG (H+L) (1:400; A11001, Life technologies, USA) served as secondary antibodies and incubated with sections at 37°C for 1 h. Similarly, testes sections from DENV-2-infected mice were also stained with mouse anti-DENV-2 polyclonal antibody followed by incubation with Alexa Fluor® 488 goat anti-mouse IgG. DAPI were used to display cell nuclei. Normal mouse testis served as control. All images were captured with a laser confocal scanning microscopy (Leica TCS SP5).

For analyzing tight junction in testes, immunofluorescence assay (IFA) was performed. anti-mouse ZO-1(1:200, Thermo Fisher, 33-9100), Ocln (1:200, Thermo Fisher, 71-1500) and Cldn-1 (1:200, Abcam, AB10598), as the primary antibodies, were applied to the sections of testis from ZIKV-infected or control mouse and incubated with the testis sections at 4°C overnight. Alexa Fluor® 594 donkey anti-rabbit IgG (H+L) (1:400; A21207, Life technologies, USA) served as the secondary antibody. Images were captured with a laser confocal scanning microscopy (Leica TCS SP5).

### Hematoxylin and eosin analyses

The ZIKV-challenged mice were euthanized by cervical dislocation at 2, 5, 8 dpi and testes were immediately fixed in Modified Davidson's Fluid solution (30 ml of 40% formaldehyde, 15 ml of ethanol, 5 ml of glacial acetic acid and 50 ml of distilled water) overnight. Testes embedded in paraffin were sectioned (5 μm in thickness) and stained with Hematoxylin and Eosin (H&E). Normal mice and DENV-2-infected mice served as control.

### Transcriptome analysis

Testes from mice with ZIKV infection were harvested at 5 dpi and subjected to KangChen Bio-tech. Inc. (Shanghai, China) for transcriptome analysis. Uninfected mice serve as control. There were three mice in each group. Briefly, after extraction of total RNA, magnetic beads with Oligo (dT) were used to isolate mRNA. The mRNA was fragmented and the fragments were enriched and purified by KAPA Stranded RNA-Seq Library Prep Kit (Illumina) to create cDNA libraries. Real-Time PCR System was used to quantify and qualify the sample libraries. Finally, the cDNA libraries were sequenced using HiSeq 2000 Sequencing System (Illumina, Inc., USA). Image processing and base recognition were conducted by Solexa pipeline version 1.8 (Off-Line Base Caller software, version 1.8). Gene expression level and the transcription level (FPKM value) were calculated by Cufflinks 2 software (v2.1.1) to screen differentially expressed genes between groups.

### Transmission electron microscope (TEM)

Testes with or without ZIKV infection were fixed with 2.5% glutaraldehyde in phosphate buffer (PB, pH7.4) for 2 h and post-fixed with 1% OsO4 in PB for 1 h. After washed three times in PB, specimens were dehydrated by a graded series of ethanol (30, 50, 70, 80, 90, 95, and 100%, 15 min at each step) and then successively infiltrated at room temperature in 1:1 mixture of absolute acetone and embedding medium for 1 h followed by 1:3 mixture of absolute acetone and embedding medium for 3 h. Then the specimens were subjected to embedding medium overnight. After embedded and ultrathin sectioned, sections were stained by uranyl acetate and alkaline lead citrate for 15 min, respectively. Specimens were observed and captured under transmission electron microscope (H-7500, HITACHI, Japan).

### Papanicolaou staining for examining morphology of sperm cells

To obtain mature sperms, cauda epididymidis of mouse were harvested and incubated in PBS at 37°C for 30 min to allow sperm to swim out. After smeared the supernatant and air-dried naturally, slices were stained by standard procedures of Papanicolaou staining according to WHO guidance (World Health Organization, 2016). Briefly, slides were first immersed in 95% ethanol for 15 min to fix and followed by sequentially immersed in 80% ethanol, 50% ethanol, purified water and stained in Harris's hematoxylin. After differentiation by hydrochloric acid ethanol, slices were sequentially immersed in 50% ethanol, 80% ethanol, 95% ethanol and stained by G-6 orange stain and EA-50 green stain. After washed by 95% ethanol and 100% ethanol, slices were mounted by neutral resins and observed under light microscope.

### Infection experiments with sertoli cells *In vitro*

Sertoli cells were challenged with ZIKV at a multiplicity of infection (MOI) of 1 for 1 h at 32°C. Afterwards, supernatants at different time-points after infection, as indicated were sampled and titers were determined by plaque assay on monolayer of Vero cells under overlay medium containing 1.2% methylcellulose. IFA was performed to detect expression of viral antigen in sertoli cells at 48 h after infection and mouse anti-ZIKV polyclonal serum served as primary antibody. Three independent experiments were performed for each time point.

### Analysis of significance

Statistical analysis was performed with SPSS 17.0. The quantitative data between two groups were compared using a *t*-test. Differences among the groups were considered to be significant at *p* < 0.05.

## Author contributions

ZS and NG performed the animal experiments and most other experiments. ZW performed the experiments on immortal sertoli cells. XC and DF maintained the cells and virus used in the experiments. DZ, HC, PW, and JA designed the experiments and analyzed the results. PW and JA prepared and revised the manuscript. JA organized the collaboration and directed the project.

### Conflict of interest statement

The authors declare that the research was conducted in the absence of any commercial or financial relationships that could be construed as a potential conflict of interest.
